# Supplementary material for: scSPLAT, a scalable plate-based protocol for single cell WGBS library preparation
Source: Sci Rep. 2022 Apr 6;12:5772. doi: 10.1038/s41598-022-09798-2 (PMC8986790; doi:10.1038/s41598-022-09798-2)

Supplementary Information

Supplementary Table S1; Oligo sequences

Supplementary Figures S1-S6

Supplementary Data S1; Sample and read alignment information, excel sheet

Supplementary Data S2; Liver cluster assignments, excel sheet

Supplementary Data S3; DMR annotations, excel sheet

Supplementary Data S4; GO:BP intersections, excel sheet

Supplementary Data S5; DMR-marker overlaps, excel sheet

Table S1. Oligo sequences

Oligo name	Sequence
SSO_H_1	5AmMC6/TTCCCTACACGACGCTCTTCCGATCTTAGCTTHHHHHHHH
SSO_H_2	5AmMC6/TTCCCTACACGACGCTCTTCCGATCTACTTGAHHHHHHHH
SSO_H_3	5AmMC6/TTCCCTACACGACGCTCTTCCGATCTGCCAATHHHHHHHH
SSO_H_4	5AmMC6/TTCCCTACACGACGCTCTTCCGATCTCGATGTHHHHHHHH
SSO_H_5	5AmMC6/TTCCCTACACGACGCTCTTCCGATCTATCACGHHHHHHHH
SSO_H_6	5AmMC6/TTCCCTACACGACGCTCTTCCGATCTTGACCAHHHHHHHH
SSO_H_7	5AmMC6/TTCCCTACACGACGCTCTTCCGATCTCAGATCHHHHHHHH
SSO_H_8	5AmMC6/TTCCCTACACGACGCTCTTCCGATCTCTTGTAHHHHHHHH
SSO_H_9	5AmMC6/TTCCCTACACGACGCTCTTCCGATCTGACGAGHHHHHHHH
SSO_H_10	5AmMC6/TTCCCTACACGACGCTCTTCCGATCTAAGGTCHHHHHHHH
SSO_H_11	5AmMC6/TTCCCTACACGACGCTCTTCCGATCTTGTAGCHHHHHHHH
SSO_H_12	5AmMC6/TTCCCTACACGACGCTCTTCCGATCTTTGGCAHHHHHHHH
SSO_H_13	5AmMC6/TTCCCTACACGACGCTCTTCCGATCTCTACTGHHHHHHHH
SSO_H_14	5AmMC6/TTCCCTACACGACGCTCTTCCGATCTCATATGHHHHHHHH
SSO_H_15	5AmMC6/TTCCCTACACGACGCTCTTCCGATCTGTATACHHHHHHHH
SSO_H_16	5AmMC6/TTCCCTACACGACGCTCTTCCGATCTACCTTCHHHHHHHH
SSO_H_17	5AmMC6/TTCCCTACACGACGCTCTTCCGATCTCTTCCAHHHHHHHH
SSO_H_18	5AmMC6/TTCCCTACACGACGCTCTTCCGATCTCGAATGHHHHHHHH
SSO_H_19	5AmMC6/TTCCCTACACGACGCTCTTCCGATCTAGCATCHHHHHHHH
SSO_H_20	5AmMC6/TTCCCTACACGACGCTCTTCCGATCTTACCGAHHHHHHHH
SSO_H_21	5AmMC6/TTCCCTACACGACGCTCTTCCGATCTCAAGGAHHHHHHHH
SSO_H_22	5AmMC6/TTCCCTACACGACGCTCTTCCGATCTCCGTAAHHHHHHHH
SSO_H_23	5AmMC6/TTCCCTACACGACGCTCTTCCGATCTATAGACHHHHHHHH
SSO_H_24	5AmMC6/TTCCCTACACGACGCTCTTCCGATCTAGGTCTHHHHHHHH
SSO_H_25	5AmMC6/TTCCCTACACGACGCTCTTCCGATCTGGAATGHHHHHHHH
SSO_H_26	5AmMC6/TTCCCTACACGACGCTCTTCCGATCTGCTTCAHHHHHHHH
SSO_H_27	5AmMC6/TTCCCTACACGACGCTCTTCCGATCTTCCTATHHHHHHHH
SSO_H_28	5AmMC6/TTCCCTACACGACGCTCTTCCGATCTTAAGGCHHHHHHHH
SSO_H_29	5AmMC6/TTCCCTACACGACGCTCTTCCGATCTAACCTGHHHHHHHH
SSO_H_30	5AmMC6/TTCCCTACACGACGCTCTTCCGATCTTTCCCGHHHHHHHH
SSO_H_31	5AmMC6/TTCCCTACACGACGCTCTTCCGATCTGATCATHHHHHHHH
SSO_H_32	5AmMC6/TTCCCTACACGACGCTCTTCCGATCTGCACTTHHHHHHHH
ss1a	5’AmMC6/GACGTGTGCTCTTCCGATCTNNNNNN/3’AmMo
ss1b	5’Phos/AGATCGGAAGAGCACACGTC/3’AmMo

Supplementary Fig. S1 Examples of TapeStation profiles. Library size profiles for pooled scSPLAT libraries

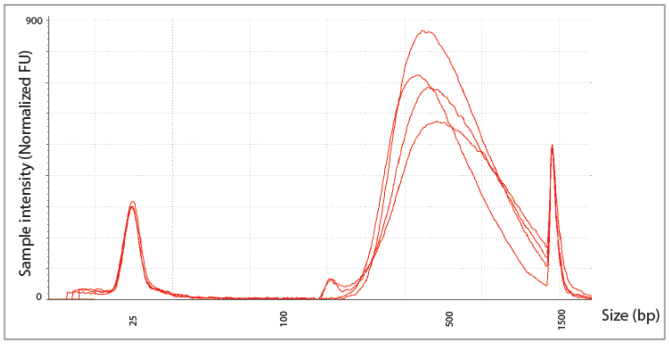

Supplementary Fig. S2 Demultiplexing profiles. A) K562 16-cell pools and B) liver nuclei 32-cell pools. The bars represent the proportion of reads assigned to each cell in the pool.

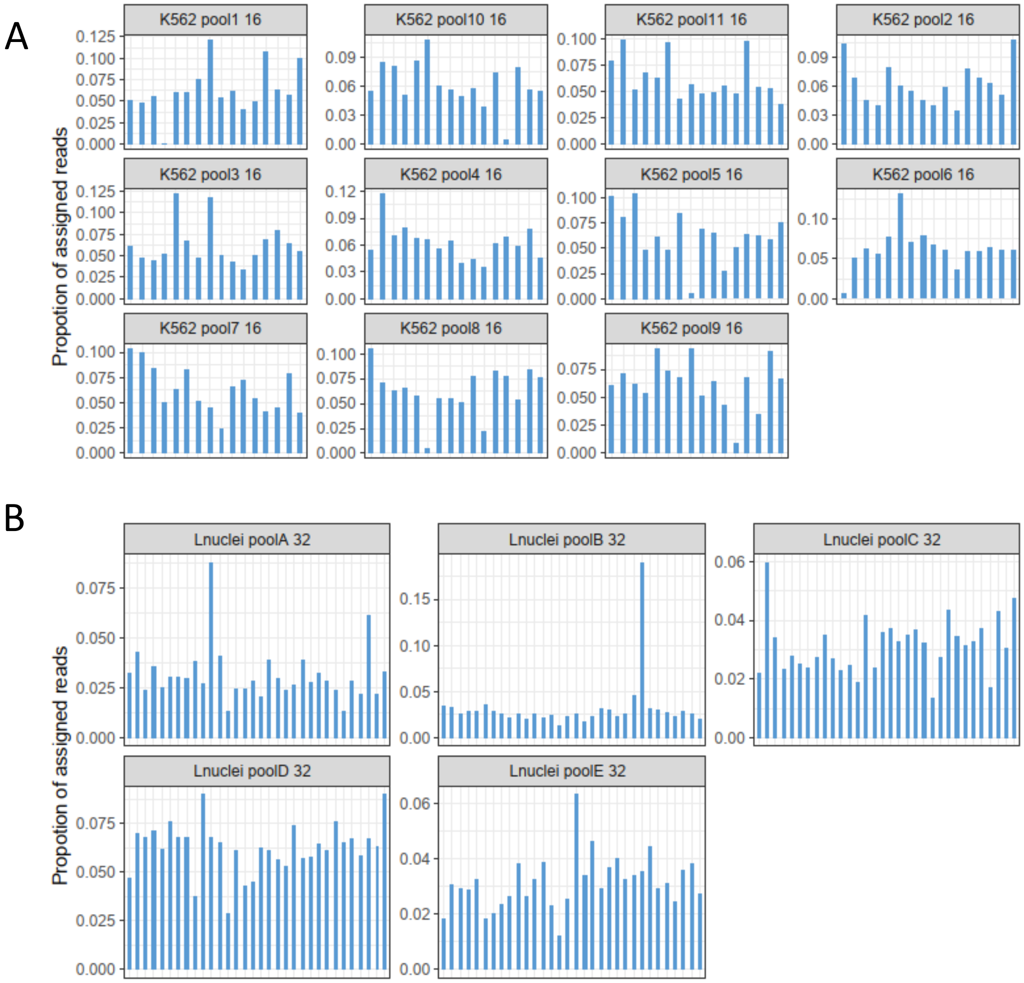

Supplementary Fig S3 K562 and liver nuclei EpiClomal Region clustering based on methylation in CpG islands. A) K562 cells colored by batch. B) K562 cells colored pool-wise**.** C) Liver nuclei colored by batch. D) Liver nuclei colored pool-wise.

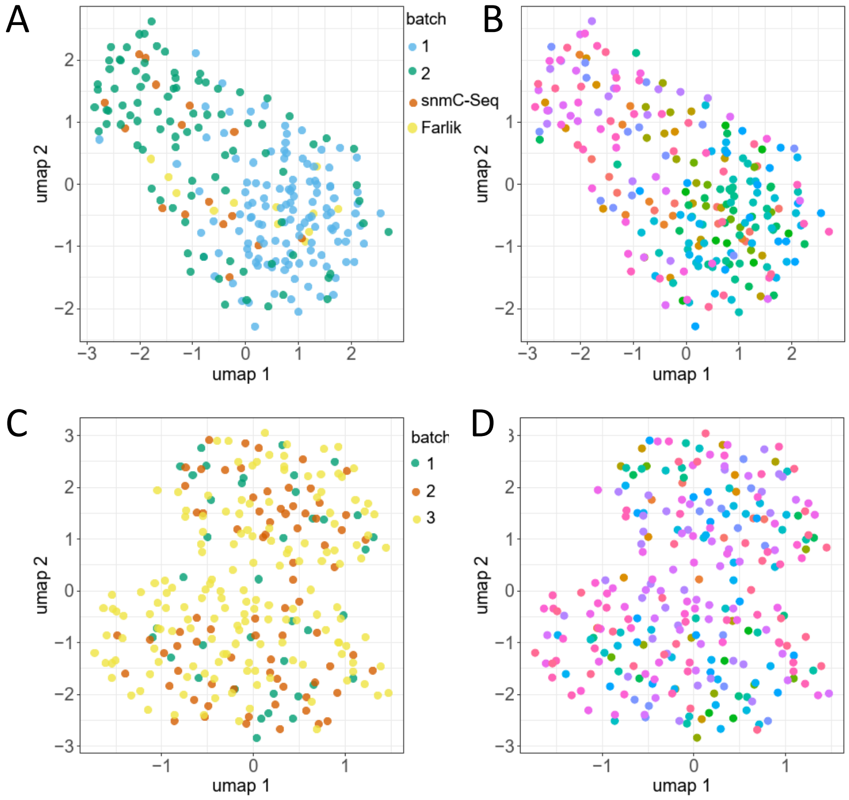

Supplementary Fig. S4 Alternative clustering of K562 cells. EpiClomal Region assigned all K562 cells to a single cluster irrespective of whether the clustering was performed based on methylation in CpG islands, gene bodies or TSS regions. A) K562 EpiClomal Region clustering based on methylation in gene bodies. Cells colored by batch. Although it appears like a small number of cells form a 'subcluster' in the UMAP plot, they were all assigned to one single cluster by EpiClomal. B) K562 EpiClomal Region clustering based on methylation in regions of 1000 bp +/- of TSS. Cells colored by batch.

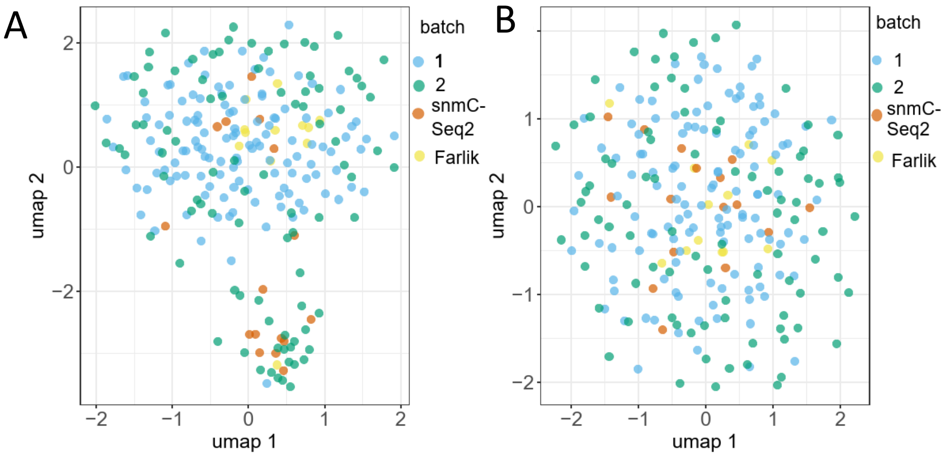

Supplementary Fig. S5 Liver nuclei QC metrics A) Number of detected CpG sites per cell vs sequencing depth B) Global methylation levels and read alignment efficiency per nuclei.

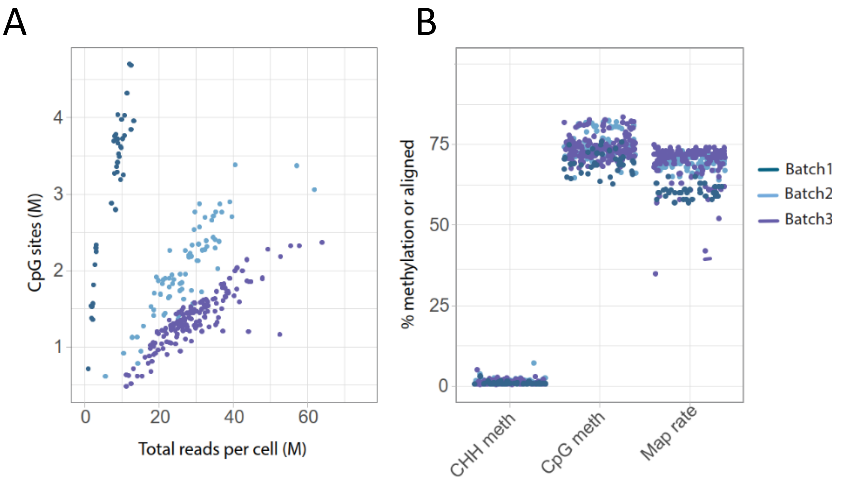

Supplementary Fig. S6 Reclustering of liver nuclei cluster 1. Clustering was perfomed on: CpG island methylation, gene body methylation and methylation across TSS regions. With the two latter EpiClomal finds one cluster only. In contrast, with CpG-island methylation EpiClomal assigns the cells to two clusters that were were intermingled in the UMAP visualization.

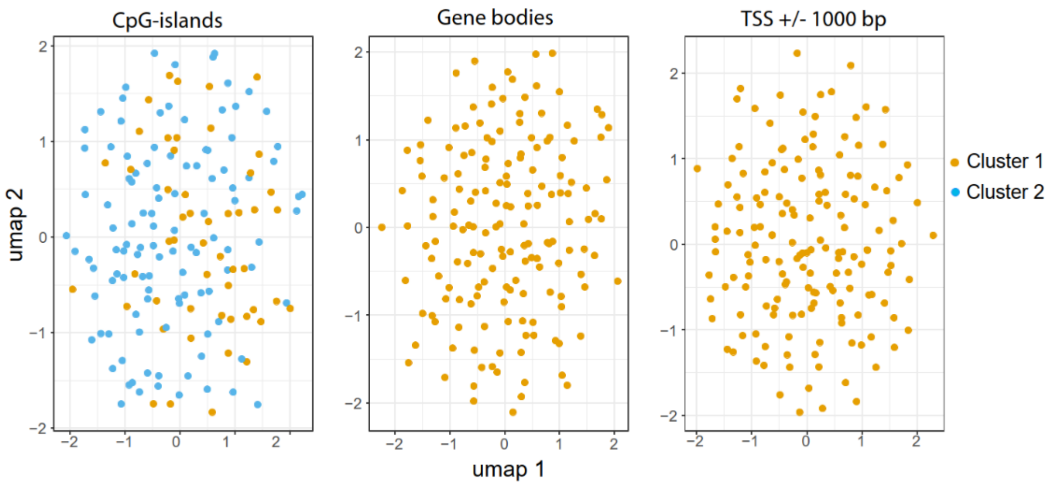

Supplement: Supplementary file 1 — Supplementary Information 1. [file 41598_2022_9798_MOESM1_ESM.pdf]
